# Integrative Analyses of Genes Associated with Hashimoto's Thyroiditis

**DOI:** 10.1155/2021/8263829

**Published:** 2021-08-28

**Authors:** Kangli Qiu, Kai Li, Tianshu Zeng, Yunfei Liao, Jie Min, Nan Zhang, Miaomiao Peng, Wen Kong, Lu-lu Chen

**Affiliations:** ^1^Department of Endocrinology, Union Hospital, Tongji Medical College, Huazhong University of Science and Technology, Wuhan 430022, China; ^2^Hubei Provincial Clinical Research Center for Diabetes and Metabolic Disorders, Wuhan 430022, China; ^3^Network and Computing Center, Huazhong University of Science and Technology, Wuhan 430022, China

## Abstract

**Objective:**

Hashimoto's thyroiditis, also known as chronic lymphocytic thyroiditis, is a common autoimmune thyroiditis, which mostly occurs in young and middle-aged women. It can be manifested as hyperthyroidism in the early stage; hypothyroidism may appear with the progression of the disease. Studies have shown that multiple factors such as heredity, environment, and autoimmunity are involved in the pathogenesis, but the specific mechanism is not clear. In our study, we tried to find key genes and potential molecular mechanisms of Hashimoto's thyroiditis to provide new ideas for the therapeutic targets of Hashimoto's thyroiditis.

**Method:**

GSE138198 and GSE54958 were downloaded from the GEO database, and two datasets were combined for analysis. The combined data were normalized to identify the differentially expressed genes (DEGs), and GO and KEGG enrichment analyses were performed. Protein-protein interaction (PPI) networks and hub genes between DEGs were identified. We also used the miRWalk database to identify regulatory miRNAs associated with expressions of DEGs.

**Result:**

We identified 182 DEGs (160 upregulated and 22 downregulated) between Hashimoto's disease patients and the healthy control group. GO analysis showed that DEGs were mostly concentrated in detection of chemical stimulus involved in sensory perception, intermediate filament cytoskeleton, and olfactory receptor activity. KEGG pathway analysis showed that DEGs were mainly related to olfactory transduction. Some members of the KRTAP family and HTR5A, KNG1, DRD3, HTR1D, TAS2R16, INSL5, TAS2R42, and GRM7 are the most important hub genes in the PPI network. In addition, we recognized that OTUD4, LLPH, and ECHDC1 were the most important hub genes in the miRNA-target gene network.

**Conclusion:**

In this study, a series of bioinformatics analyses of DEGs were performed to identify the key genes and pathways associated with Hashimoto's thyroiditis. These genes and pathways provide a more detailed understanding of the pathogenesis of Hashimoto's disease and provide new ideas for the therapeutic targets of Hashimoto's thyroiditis.

## 1. Introduction

Hashimoto's thyroiditis, also known as chronic lymphocytic thyroiditis, is an autoimmune thyroid disease, first described by Hiroshi Hashimoto in 1912. He reported on four patients with chronic thyroid disease, which he called thyroid lymphoma, which was characterized by diffuse lymphocytic infiltration, germinal centers, parenchymal atrophy, fibrosis, and eosinophilic changes in some thyroid follicular cells [1]. The incidence of Hashimoto's disease is about 0.3-1.5 cases per 1000 people per year [2], and it is gradually increasing. The incidence of women is 5-10 times that of men, and it increases with age (peak age is between 45 and 65 years) [3]. However, it is worth noting that the disease can be diagnosed in patients of any age, including children [4]. HT is genetically susceptible, many of which are immune-related and thyroid-specific genes that confer disease susceptibility. It has been found that the increase in the incidence of HT is related to environmental factors, including improved sanitation, increased dietary iodine intake, new treatment modalities, and chemical agents. Other unchangeable predisposing factors include stress, climate, age, and gender [5]. The diagnosis of Hashimoto's thyroiditis is based on clinical features, typical ultrasound patterns, cytological examination of lymphocytic infiltration, and positive serum antibodies to thyroid antigens (including thyroid peroxidase and thyroglobulin) [6]. HT is usually associated with other autoimmune diseases, such as hair loss, leukoplakia, celiac disease, and type 1 insulin-dependent diabetes. Thyroid function at the time of HT may be variable, from a short period of hyperthyroidism to obvious hypothyroidism. If there is obvious hypothyroidism, treatment with L-thyroxine should be started immediately [7].

At present, Hashimoto's thyroiditis is believed to be related to multiple factors such as genetic susceptibility, environmental factors, and immune disorders [8], and cellular and humoral immunity plays a key role in the development of the disease. In the etiology of Hashimoto's thyroiditis, overstimulated CD4+ cells play the most important role. Recent studies have shown that newly discovered cells such as regulatory T cells (CD4+CD25+HighFoxP3+) or Th17 (CD4+IL-17+) play an important role in inducing autoimmune diseases [9]. Programmed cell death also plays an equally important role in the onset and development of hypothyroidism [10–12].

Until now, several loci have been found to be associated with Hashimoto's thyroiditis, such as HLA-DR, immunoregulatory genes (CD40, FoxP3, CD25, CTLA-4, and PTPN22), and thyroid-specific genes (thyrotropin (TSH) receptors and thyroglobulin) [13–16]. By analyzing the microarray data of GSE29315, Zheng et al. reported that 10 hub genes and interferon-*γ*, IFN-*α*, IL-6/JAK/STAT3, and inflammatory pathways may promote the occurrence and development of HT [17]. However, no studies have reported the possible regulatory mechanism of microRNAs related to the occurrence of Hashimoto's thyroiditis.

In our study, GSE138198 and GSE54958 were analyzed by the bioinformatics method, and the two datasets were combined for analysis, and the combined data were normalized to identify the differentially expressed genes (DEGs) for functional enrichment analysis. Protein-protein interaction (PPI) analysis and regulatory miRNAs were related to DEG prediction. Through these analyses, we expect to provide novel insights into the pathogenesis of Hashimoto's thyroiditis and provide a more detailed molecular mechanism for the development of Hashimoto's thyroiditis.

## 2. Materials and Methods

### 2.1. Data Selection

The GSE138198 and GSE54958 datasets were downloaded from the GEO website, and the GPL6244[hugene-1_0-st]Affymetrix human gene 1.0 ST array [transcript (gene version)] was used for the two expression matrices. The GSE138198 dataset included 13 samples of Hashimoto's thyroiditis, 3 normal thyroid samples, 12 samples of thyroid papillary carcinoma, and 8 samples of thyroid papillary carcinoma with Hashimoto's thyroiditis. The GSE54958 dataset included 7 normal thyroid samples and 25 thyroid papillary carcinoma samples. Since these two datasets are from the same chip platform, they can be combined and analyzed, and the combined data is normalized.

### 2.2. Data Processing

The GSE138198 and GSE54958 raw datasets were evaluated using the limma R package, and the two datasets were merged for joint analysis. We firstly corrected the data, obtained the expression matrix dataset of experimental requirements in the form of a subset, and then extracted the corresponding clinical information for the classification of subsequent samples according to the data samples of the expression matrix. Finally, the two datasets GSE138198 and GSE54958 were combined according to the gene ID. The combined samples were normalized to exclude the influence caused by the batch effect. By data processing, we finally get the normal thyroid samples (7 cases) as the control group with Hashimoto's thyroiditis samples as the treatment group (13 cases) by using∣log2FC | >2 and adjusted *P* < 0.05to identify Hashimoto's thyroiditis DEGs.

### 2.3. Functional and Pathway Enrichment Analysis

Gene Ontology (GO) term enrichment analysis and Kyoto Encyclopedia of Genes and Genomes (KEGG) pathway analysis, based on R software, were applied for the identification of pathways in which DEGs were significantly enriched. GO enrichment analysis includes three parts: biological process, molecular function, and cellular component. The http://org.hs.eg.db database file on the Bioconductor platform is used for the database, which contains 28 mainstream data files. We performed GO enrichment analysis of Hashimoto's thyroiditis differentially expressed genes [18] and KEGG pathway enrichment analysis [19] to analyze the biological processes and key pathways in which the differentially expressed genes were mainly involved, and *P* < 0.01 for GO enrichment analysis was the inclusion criteria, and *P* < 0.05 for KEGG enrichment pathway analysis was the inclusion criteria.

### 2.4. PPI Network Construction

We used the online tool Search Tool for the Retrieval of Interacting Genes (STRING V-11.0, https://string-db.org/) database to evaluate PPI information. In order to evaluate the interaction relationship between DEGs, DEGs were mapped to STRING, and the interaction relationship between DEGs was screened at the protein level to construct a PPI network with upregulation and downregulation of DEGs. Then, PPI network visualization is constructed by using Cytoscape software. Hub genes of the PPI network were screened by CytoHubba in Cytoscape, and the top 20 hub genes were selected for analysis.

### 2.5. MicroRNA-Target Gene Network Prediction

In the disease state, gene expression is affected by microRNA through the posttranscriptional control. In this study, the miRWalk database (http://miRWalk.umm.uni-heidelberg.de/) is used to search for miRNAs related to DEGs. miRWalk is a publicly available comprehensive resource, a comprehensive miRNA-target gene database, including predicted and experimentally verified microRNA- (miRNA-) target interaction pairs, including Human, Mouse, Rat, Dog, and Cow [20, 21]. The miRNA binding sites on the full-length sequence of the gene are not only recorded but also compared with the prediction binding information collection of the existing 12 miRNA-target prediction programs (DIANA-microTv4.0, miRanda-rel2010, DIANA-microT-CDS, miRDB4.0, miRmap, miRNAMap, miRBridge, doRiNA, i.e., PicTar2, RNAhybrid2.1, PITA, RNA22v2, and TargetScan6.2).

## 3. Results

### 3.1. Identification of DEGs in Hashimoto's Thyroiditis (HT)

**Figure 1:**
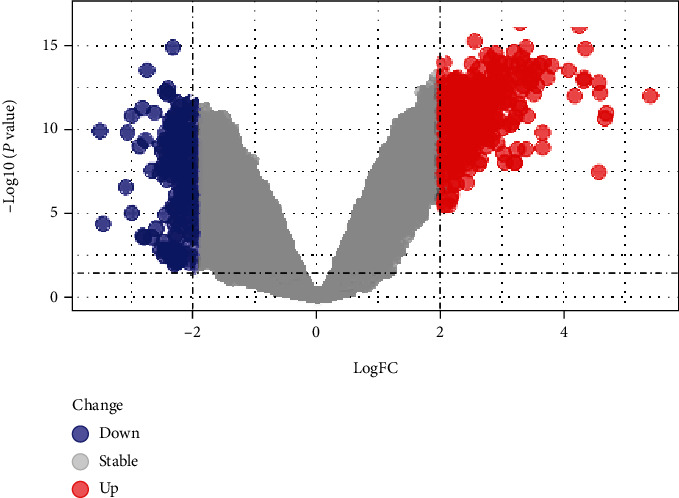
Volcano map of DEGs.

**Figure 2:**
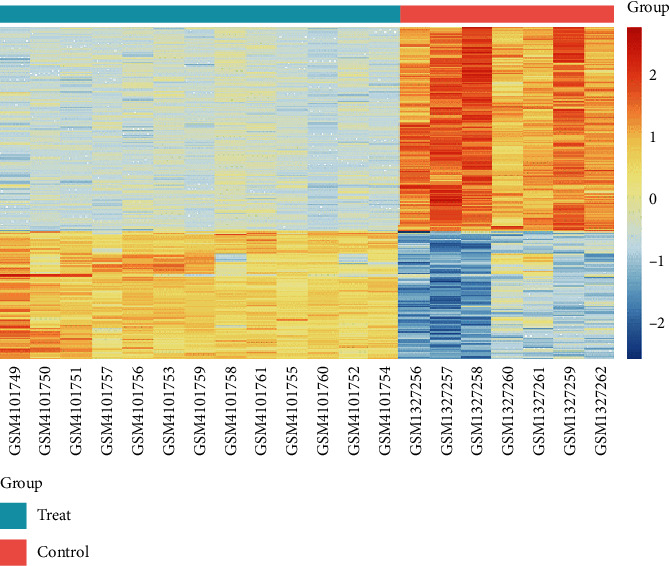
Heat map of DEGs.

We identified 182 DEGs in HT patients, with a total of 160 upregulated genes and 20 downregulated genes compared with healthy controls. We drafted a volcano map of DEGs (Figure 1) and a hierarchical clustering heat map of the differentially expressed genes (Figure 2). The results showed that there were good differences in these DEGs between HT patients and healthy controls. OR2J3 and IL7R were identified as the most significantly upregulated and downregulated genes in HT patients, respectively.

### 3.2. Functional and Pathway Enrichment Analysis

**Figure 3:**
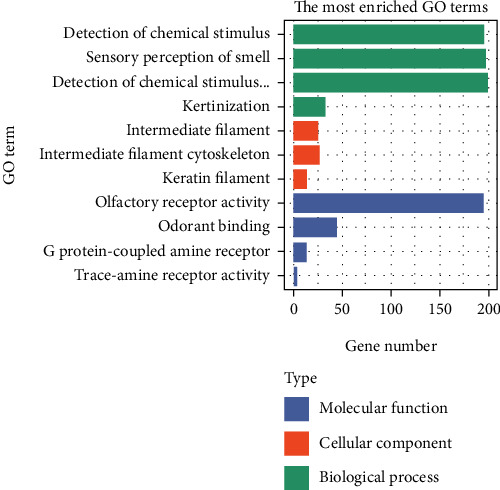
The results of GO of DEGs.

**Figure 4:**
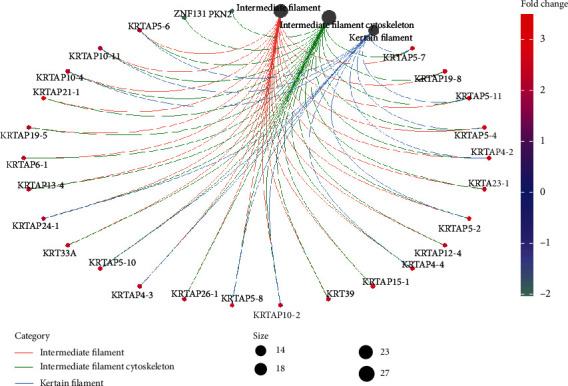
Pathway diagram of cellular components (CC).

We used R language to analyze the differentially expressed genes using the http://org.Hs.eg.db database file on the Bioconductor platform; a total of 11 GO in terms of biological processes, molecular functions, and cellular components (Figures 3 and 4) and 1 KEGG (Table 1) were identified. BP was mainly concentrated in detection of chemical stimulus involved in sensory perception, detection of chemical stimulus, and sensory perception of smell involved in sensory perception and keratinization; CC was mainly concentrated in intermediate filament, intermediate filament cytoskeleton, and keratin filament; MF was mainly concentrated in odorant binding, olfactory receptor activity, trace-amine receptor activity, and G protein-coupled amine. In addition, KEGG pathway analysis showed that DEGs are closely related to olfactory transduction.

### 3.3. PPI Network and Hub Gene Identification

**Figure 5:**
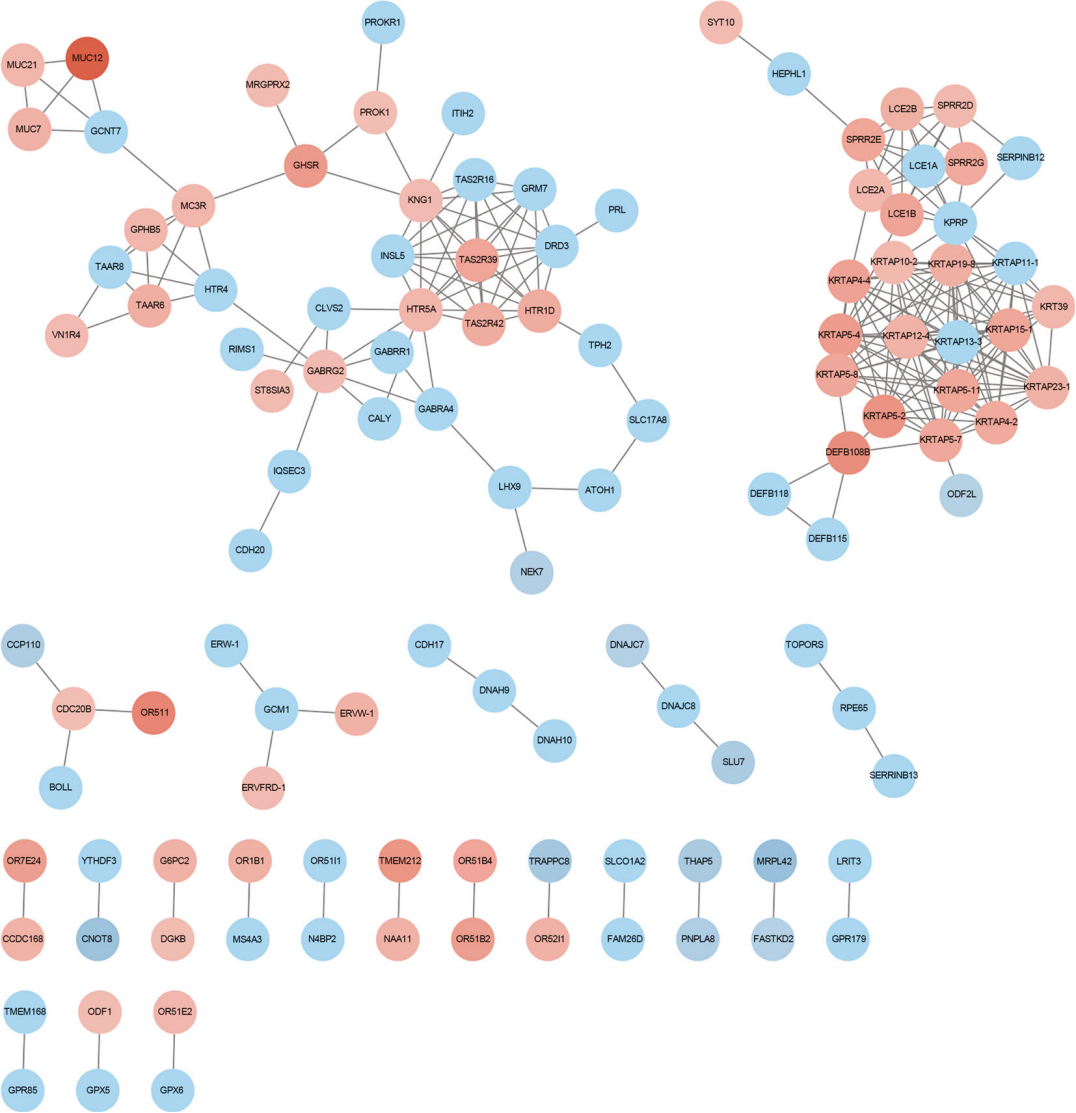
PPI network of the DEGs.

**Figure 6:**
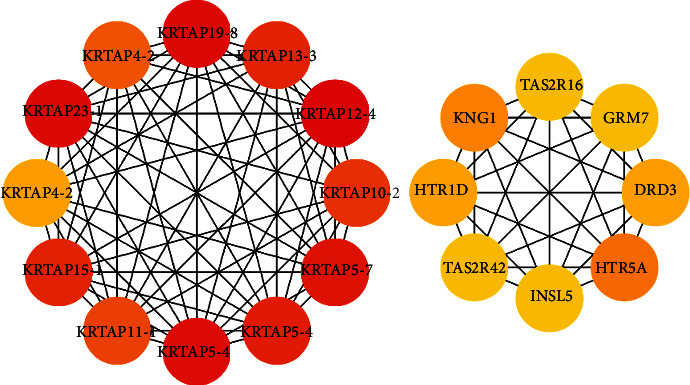
Hub genes in the PPI network of the DEGs.

We submit DEGs to the STRING database to obtain PPI data. We used Cytoscape 3.8.2 to construct a PPI network (Figure 5) and identified the first 20 genes as hub genes (Figure 6). Among them, the first 12 belong to the KRTAP family of genes, and the last 8 are HTR5A, KNG1, DRD3, HTR1D, TAS2R16, INSL5, TAS2R42, and GRM7, all of which are upregulated genes.

### 3.4. MicroRNA-DEG Regulatory Network Analysis

**Figure 7:**
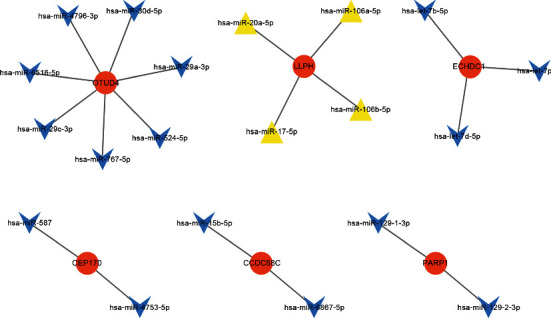
MicroRNA-DEG regulatory network. Blue and yellow diamonds stand for miRNAs, and orange nodes stand for DEGs.

Figure 7 shows the miRNAs of DEGs, 7 downregulated miRNAs regulate OTUD4, 4 upregulated miRNAs regulate LLPH, and 3 downregulated miRNAs regulate ECHDC1.

## 4. Discussion

HT was first discovered and described by Hiroshi Hashimoto. It is an autoimmune thyroid disease [22] and is related to multiple factors such as genetic susceptibility, environmental factors, and immune disorders [19, 20]. With the progression of the disease, hypothyroidism may occur, and alternative therapies are used [23–27]. It is of great significance to understand the molecular mechanism of HT. We downloaded and analyzed data information of 13 HT patients and 7 healthy controls from the GEO database. We identified a total of 182 DEGs, including 160 upregulated DEGs and 22 downregulated DEGs. Among 182 DEGs, we noticed that IL7R is the most downregulated gene in HT patients. The protein encoded by this gene is the receptor for interleukin 7 (IL-7). This protein plays a key role in V(D)J recombination during lymphocyte development and is essential for normal T cell development and homeostasis. The function of this receptor requires the interleukin 2 receptor gamma chain, which is a gamma chain shared by a variety of cytokine receptors. The defection of this gene may be related to severe combined immunodeficiency (SCID) [28–34]. Studies have shown that IL7R signaling is essential for the development of thymocytes [35, 36]. A Japanese study found that there were no prominent differences in expression levels of IL7R and common cytokine receptor *γ*-chain between the CLTH, control, and TL cases, but the ISH method showed that compared with CLTH, the number of cells expressing IL-7 in TL increased significantly, but the study did not use the ISH method to compare the expression levels of IL-7 in CLTH and control cases [37]. The role of IL7R in HT needs to be further studied. Among the increased DEGs, OR2J3 is the most upregulated gene in HT, which encodes a G protein-coupled receptor (GPCR) that functions as an olfactory receptor. Olfactory receptors interact with odor molecules in the nose, triggering neuronal responses that trigger the sense of smell. This gene is located in a cluster on chromosome 6 similar to the gene encoding the olfactory receptor [38, 39]. There is no research showing that OR2J3 is related to HT, and it is a new biomarker for the occurrence of HT.

In our research, the most important GO BP term for DEGs is detection of chemical stimulus involved in sensory perception. OR1N1, OR5M10, and OR51V1 are new biomarkers for the development of HT. The most important GO MF term is olfactory receptor activity. The most important GO CC term is intermediate filament cytoskeleton. KRTAP5-7, KRTAP19-8, and KRTAP5-11 are new biomarkers developed by FT1D. There were significant changes in OR4N4 gene expression in human thyroid epithelial cell lines after exposure to high-dose gamma radiation [40], and OR8B12, OR56A3, and OR4N4 are all new biomarkers for the development of HT.

Olfactory transduction is the most important KEGG pathway of DEGs, and OR7G1, OR8I2, OR9K2, OR2M5, and OR52N1 are new biomarkers of HT progression.

In this study, some members of the KRTAP family and HTR5A, KNG1, DRD3, HTR1D, TAS2R16, INSL5, TAS2R42, and GRM7 are considered to be the hub genes in the PPI network. Keratin-associated proteins (KRTAP) are the major components of hair [41]. KNG1 is believed to be related to the occurrence of thyroid cancer [42]. INSL5 and INSL3 are both members of the insulin-like hormone superfamily. Research indicates that the INSL3 hormone is upregulated in neoplastic and hyperplastic human thyrocytes, suggesting that the INSL3 isoforms may serve as markers for neoplastic and hyperplastic human thyrocytes [43], but there is no report on the correlation between INSL5 and HT. TAS2Rs control the production of thyroid hormones, and this regulation may be related to the susceptibility to thyroid diseases. TAS2R agonists can inhibit basic and TSH-dependent iodide outflow. TAS2R may mediate a protective response to excessive intake of toxic substances, as a new drug target for the treatment of hypothyroidism or hyperthyroidism. Genetic variants in the TAS2R receptor may change the risk of papillary thyroid cancer (PTC) [44, 45]. They are new biomarkers for the occurrence of HT.

OTUD4, LLPH, and ECHDC1 are the three most important target genes in the miRNA-target gene regulatory network. OTUD4 is the fifth frequently mutated gene in thyroid cancer, and its mutation rate in the thyroid is 2% [46]. LLPH and ECHDC1 are new biomarkers for the development of HT.

When we searched the articles, we found that there was a bioinformatics analysis on HT which used thyroid physiological hyperplasia (TPH) as a control group, not the healthy thyroid tissue, so there is no similarity with our conclusions [17]. There are no other reports about the discovery of HT hub genes and pathways. The results of our bioinformatics analysis will help to research HT in the future.

Our study has certain limitations. We only found 2 matrix datasets about HT in the GEO database. One of them is about HT and PTC, and there is no healthy thyroid sample as a control, so we did not use it. The GSE138198 dataset we used includes 13 cases of HT and 3 healthy thyroid samples. Since there are fewer healthy samples, we combined the 7 healthy thyroid samples in the GSE54958 dataset from the same chip platform and the 3 healthy thyroid samples in the GSE138198 dataset for analysis together and normalized the combined data to expand the healthy thyroid group samples. Given the potential analysis bias, we will conduct further molecular biology experiments in the follow-up work to verify the function of hub genes at the cell or specimen level.

## 5. Conclusions

Our study provides a comprehensive DEG bioinformatics analysis for finding molecular mechanisms related to the progression of HT. We discovered some meaningful genes to study the molecular mechanism of HT progression. Further experiments at the cell or specimen level will be conducted to confirm the role of these DEGs in the progression of HT.

## Figures and Tables

**Table 1:** The results of KEGG of DEGs.

ID	Description	*P* value	Count	Symbol
hsa04740	Olfactory transduction	1.44*E*-119	103	OR1N1/OR5M10/OR51V1/OR8B12/OR56A3/OR4N4/OR5A2/OR2F2/OR1C1/ OR51G2/OR5I1/OR6N2/OR4K13/OR8B8/OR51B2/OR2T27/OR52B6/OR1A2/ OR5D16/OR6V1/OR6C75/OR4F6/OR1D5/OR4C13/OR8D2/OR5AP2/OR6C3/ OR52B4/OR52A5/OR4K15/OR10R2/OR8G1/OR4K1/OR13F1/OR1S1/OR2K2/ OR7E24/OR6B1/OR1S2/OR4C16/OR1F1/OR8K3/OR6Y1/OR1G1/OR4N5/OR6K2/ OR4L1/OR6C74/OR6K6/OR5R1/OR4K2/OR1L3/OR5F1/OR2G3/OR2J3/OR6P1/ OR1J4/OR8K5/OR51B4/OR13C4/OR6T1/OR52H1/OR2M4/OR52E2/OR5D14/ OR9A2/OR7G1/OR8A1/OR56A1/OR2A12/OR5B21/OR2L2/OR51F2/OR4K5/ OR10A7/OR10K1/OR2T1/OR51B6/OR52E4/OR6X1/OR4N2/OR2Z1/ OR51G1OR14A16/OR6C76/OR5T2/OR51T1/OR8K1/OR10G7/OR4C45/OR2L8/ OR8H2/OR1D2/OR4A47/OR5B2/OR14I1/OR51L1/OR9Q2/OR14J1/OR56A4/ OR4K14/OR52J3/OR51A4
hsa00920	Sulfur metabolism	0.134316817	1	IMPAD1
hsa03410	Base excision repair	0.379147613	1	PARP1
hsa00640	Propanoate metabolism	0.388069674	1	ECHDC1
hsa05340	Primary immunodeficiency	0.422504487	1	IL7R

## Data Availability

The data used to support the findings of this study are included within the article.
